# Supplementary material for: Independent Responses of Photosynthesis and Plant Morphology to Alterations of PIF Proteins and Light-Dependent MicroRNA Contents in Arabidopsis thaliana pif Mutants Grown under Lights of Different Spectral Compositions
Source: Cells. 2022 Dec 9;11(24):3981. doi: 10.3390/cells11243981 (PMC9776988; doi:10.3390/cells11243981)
Supplement: Supplementary file 1 [file cells-11-03981-s001.zip › cells-2012863-supplementary.pdf]

Table S1. Primers for qRT-PCR analysis

	Gene Bank ID	Gene description	Gene	Primer ′5-3′	
				forward	reverse
2	NM_001203404.1	Squamosa promoter binding protein-like 7	*SPL7*	ACGTTCGCTGTCTGTTTTGC	AGCTCTGTTGAACACAATCTCA
3	O04379	Argonaute 1	*Ago1*	TAGGGGCTGGAGGTCAAAGA	CGGAATATACCTAGAGGTCGGC
4	NM_119618.3	cryptochrome-interacting basic-helix-loop-helix 1	*CIB1*	ATGCGACAAGATCACAGGCA	TTGAGGCAACCTCTTTGGCA
5	Q8GZM7	Phytochrome interacting factor 1	*PIF1*	GAATCCGGTTTAACACGGCG	ATCGACGTTCGTCTTCTCCG
6	NM_001202630.2	Phytochrome interacting factor 3	*PIF3*	GCGCGTATAATCGGAAGGGA	ATCGACGTTCGTCTTCTCCG
7	NM_129862.3	Phytochrome interacting factor 4	*PIF4*	ATGGCGAGATGGACAAGTGG	GTC GGGTTCGAATGGGTCTT
8	NM_001335664.1	Phytochrome interacting factor 5	*PIF5*	CAGCTCGCTAGGTACATGGG	GCC GGAGATCCAAATCCCAA
9	NM_001037040.3	Phytochrome interacting factor 7	*PIF7*	GCGTCTTTTGAATCCGGTCG	GACTCGTTGTGGAATCGCTGC
10	F4I3V6	Short hypocotyl in white light 1	*SHW1*	AAGCATCGCGACGGATTTTG	CACAGTTCCTCCTAGATTGCATA
11	P42774	G-box-binding factor 1	*GBF1*	TCCCATCCCCAGTTGGATCT	GATGAGCATAGACTGCCCCC
12	Q8S4Q6	Far-red elongated hypocotyl 1	*FHY1*	TGCCAATGTTGCTTGTTCAGA	GTCTTCACCGCATGATCAG
13	Q9LIE5	Far-red elongated hypocotyl 3	*FHY3*	AACGATTTGTGCGAACGAGC	TGGTTGTCCTCCTCCACAGA
14	Q9FE22	Transcription factor HFR1	*HFR1*	GATGCCATCGCCGCTAATTC	ACCGTGAAGAGACTGAGGAGA
15	NM_121888.3	Squamosa promoter-binding-like protein 7	*SPL7*	GGATCGGGTTAGGAAGCGAG	TCAAGCACAACAAAGCTGGC
16	NM_099986.4	Endoribonuclease Dicer homolog 1	*DCL1*	ATGCAGAGGTATTATCGATGTCT	CAGGTAGGCCTTTGCAGGAT
17	NM_001203853.2	Small RNA 2'-O-methyltransferase	*HEN1*	CACCCCAAGCTCCTGATTGT	CCGAGCTTTGATGCCCATTG
18	NM_100842.4	Double-stranded RNA-binding protein 1	*HYL1*	ACACAGGAGCTGCAACAAGA	GCGGGATAGCTGCCTGTATT
19	NM_001036427.3	Arabidopsis thaliana Actin 1	*Actin1*	TTAGCAACTGGGATGACATGGA	CCTGAATGGCAACATACATAGCA
20	MIMAT0000178	ath-miR160a-5p	MIR160	TGCCTGGCTCCCTGTATGCA	
21	MIMAT0031879	ath-miR165a-5p	MIR165	GGAATGTTGTCTGGATCGAGG	
22	MIMAT0001016	ath-miR319c	MIR319	TTGGACTGAAGGGAGCTCCTT	
23	MIMAT0031908	ath-miR396a-3p	MIR396	GTTCAATAAAGCTGTGGGAAG	
24	MIMAT0000184	ath-miR163	MIR163	TTGAAGAGGACTTGGAACTTCGAT	
25	MIMAT0001003	ath-miR402	MIR402	TTCGAGGCCTATTAAACCTCTG	
26	MIMAT0000198	ath-miR168a-5p	MIR168	TCGCTTGGTGCAGGTCGGGAA	
27	MIMAT0000203	ath-miR172a	MIR172	AGAATCTTGATGATGCTGCAT	
28	MIMAT0004252	ath-miR833-5p	MIR833	TGTTTGTTGTACTCGGTCTAGT	
29	MIMAT0004243	ath-miR827	MIR827	TTAGATGACCATCAACAAACT	
30	MIMAT0031887	ath-miR170-5p	MIR170	TATTGGCCTGGTTCACTCAGA	universal reverse primer
31	MIMAT0031888	ath-miR171a-5p	MIR171	TATTGGCCTGGTTCACTCAGA	
32	MIMAT0032014	ath-miR472-5p	MIR472	ATGGTCGAAGTAGGCAAAATC	
33	MIMAT0031880	ath-miR166a-5p	MIR166	GGACTGTTGTCTGGCTCGAGG	
34	MIMAT0000196	ath-miR167a-5p	MIR167	TGAAGCTGCCAGCATGATCTA	
35	MIMAT0004302	ath-miR858a	MIR858	TTTCGTTGTCTGTTCGACCTT	
35	MIMAT0031865	ath-miR156a-5p	MIR156	TGACAGAAGAGAGTGAGCAC	
36	MIMAT0031870	ath-miR157a-5p	MIR157	TTGACAGAAGATAGAGAGCAC	
37	MIMAT0000946	ath-miR397a	MIR397	TCATTGAGTGCAGCGTTGATG	
38	MIMAT0031912	ath-miR398c-5p	MIR398	AGGGTTGATATGAGAACACAC	
39	MIMAT0031915	ath-miR408-5p	MIR408	ACAGGGAACAAGCAGAGCATG	
40	NM_001344924.1	U6 snRNA phosphodiesterase-like protein	U6	AGAGAGTTCCACATAAGCTTAGGA	
